# Supplementary material for: Lightness induction enhancements and limitations at low frequency modulations across a variety of stimulus contexts
Source: PeerJ. 2020 Apr 23;8:e8918. doi: 10.7717/peerj.8918 (PMC7183748; doi:10.7717/peerj.8918)
Supplement: Supplemental Information 1 — Readme documentation details the dataset structure. [file peerj-08-8918-s001.zip › VinkeYazdanbakhsh2019_CSV/VinkeYazdanbakhsh2019_README CSV.docx]

Description of contents for 9 csv data file in **VinkeYazdanbakhsh2019_CSV.zip**

9 stat CSV files, 1 per participant (all DSM trials)

|

| |- ***respAmp*** : measurements(4) x [stim(3) x block repetitions(1 or 2)]

| |- ***stims*** : measurements(4) x [stim(3) x block repetitions(1 or 2)]

| |- ***freq*** : measurements(4) x [stim(3) x block repetitions(1 or 2)]

9 dynam CSV files, 1 per participant (CSM trials)

|

| |- ***respAmp*** : meas(4/8) x freq x stim

| |- ***stims*** : meas(4/8) x freq x stim

| |- ***freq*** : meas(4/8) x freq x stim

***respAmp:*** Participant response amplitude logged at end of each trial (percent change from mean luminance).

***stims:*** Coding for stimulus type.

***static***: Simultaneous Contrast (1), Checkerboard (2), Münker-White (3)

***dynam***: Simultaneous Contrast (1), Checkerboard (2), Münker-White flanker (3), Münker-White assimilator (4)

***freq:*** Coding for frequency condition.

***static***: 0 Hz - manual switching

***dynam***: 0.25, 0.5, 1.0, & 2.0 Hz.
